# Oxidative stress, melatonin level, and sleep insufficiency among electronic equipment repairers

**DOI:** 10.4103/0019-5278.75692

**Published:** 2010

**Authors:** Mohamed El-Helaly, E. Abu-Hashem

**Affiliations:** Department of Public Health and Preventive Medicine, Faculty of Medicine, Mansoura University, Mansoura, Egypt; 1 Department of Clinical Pathology, Faculty of Medicine, Mansoura University, Mansoura, Egypt

**Keywords:** Electronic equipment, electromagnetic field, malondialdehyde, melatonin, sleep

## Abstract

**Background:**

Exposure to extremely low frequency electromagnetic field (ELF-EMF), especially among electronic equipment repairers may induce oxidative stress and affect sleep quality.

**Aims:**

This study was carried out to (a) investigate the effect of exposure to ELF-EMF on the malondialdehyde (MDA) levels among electronic equipment repairers as an indicator of oxidative stress; and melatonin hormone levels; and (b) to study the prevalence of sleep insufficiency among electronic equipment repairers exposed to ELF-EMF.

**Materials and Methods:**

A cross-sectional study was carried out on 50 electronic equipment repairers at high risk of exposure to ELF-EMF, and a matched control group at lower risk of exposure to ELF-EMF. All the participants completed a self-administered questionnaire about medical and occupational histories; and sleep sufficiency. The plasma melatonin and MDA levels of the study subjects were assessed.

**Results:**

The mean level of serum melatonin in the electronic equipment repairers was lower than that of the controls (*P* < 0.01). Moreover, serum MDA mean level of the electronic equipment repairers was higher than that of the controls (*P* < 0.01). Sleep insufficiency was more frequent among electronic equipment repairers (18.00%) in comparison with the controls (8.70%) (*P* > 0.05)

**Conclusion:**

The electronic equipment repairers, exposed to ELF-EMF, are at a risk of oxidative stress and sleep insufficiency, which could be explained by lower plasma melatonin levels and higher MDA levels. Health education about the hazards of ELF-EMF, shortening of exposure time per day, and taking antioxidant vitamins should be done to ameliorate the oxidative effect of EMF on those workers.

## INTRODUCTION

Most devices that have electric wires such as electric motors and electronic equipment are potential sources of extremely low frequency-electromagnetic fields (ELF-EMFs).[1] ELF-EMFs are usually defined to include 1–300 Hz, but the most important are the 50–60 Hz fields.[2] Electronic equipment repairers adjust, install, and repair electrical and electronic equipment, such as computer hardware, radio, and television equipment, tape recorders, video cassette recorders, and other video-display units (VDUs) as computer monitors.[3]

Extremely low frequency (ELF) fields at 50 or 60 Hz, which are emitted from VDUs come from the power supply, transformers, and the vertical deflection coils. Weak signals at higher radio frequencies (RF) come from the VDUs’ interior electronic circuitry and signals received from the computer. Very low-energy X-rays are produced inside the cathode ray tube, but the glass screen is thick enough to completely absorb them before they escape from the VDU.[4] According to National Institute for Occupational Safety and Health (NIOSH, 1996),[5] the range of magnetic field exposures averaged over a workday for TV repairers was 0.6–8.6 mG. However, other studies reported that radio and television assemblers and repairers were exposed to 1–1.9 mG.[6,7]

Many studies indicated that reactive oxygen species (ROS), such as superoxide anion, which is predominantly generated by the mitochondria, hydrogen peroxide produced from superoxide anion by the action of superoxide dismutase, and peroxynitrite, generated by the reaction of superoxide anion with nitric oxide have been implicated in tissue injury. The main ROS that have to be considered are the oxygen species.[8,9] When the balance of antioxidants is outweighed by excessively produced ROS as a result of overproduction of oxygen radicals, inactivation of detoxification systems, consumption of antioxidants, and failure to adequately replenish antioxidants in tissues—these endogenous antioxidative defence systems—are likely to be perturbed. It has been shown in numerous studies that ROS are directly involved in oxidative damage of cellular macromolecules, such as lipids, proteins, and nucleic acids in the tissues. Malondialdehyde (MDA) is a breakdown product of the major chain reactions leading to oxidation of polyunsaturated fatty acids and thus serves as a reliable marker of oxidative stress.[9,10]

Melatonin is a powerful, endogenously produced scavenger of ROS.[11] Many studies reported that ELF-EMF exposure from electronic equipment and VDT may decrease melatonin level, so exposure to ELF-EMF could decrease melatonin levels in the body leading to imbalance between the oxidant and the antioxidant agents that may lead to oxidative stress.[9,10,12,13]

Moreover, research into the possible effects of magnetic fields on human melatonin secretion is important from a public health perspective, because alterations in human melatonin secretion are likely to lead to clinical disorders, including sleep and mood disturbances that can be related to desynchronization of circadian rhythms.[14,15]

However, only limited researches were done on the health effects of exposure to ELF-EMF among electronic equipment repairers, so this study was carried out to (a) investigate the effect of exposure to ELF-EMF among electronic equipment repairers on MDA as an indicative of oxidative stress; and melatonin hormone levels; and (b) to study the prevalence of sleep insufficiency among electronic equipment repairers exposed to ELF-EMF.

## MATERIALS AND METHODS

### Subjects

The study was conducted in Mansoura city in Egypt during the period March–May 2009. We recruited 63 electronic equipment repairers from Mansoura city; however, three workers were excluded as they lived near high voltage power lines and mobile base stations. Then, from the remaining 60 workers, 50 electronic equipment repairers were randomly selected and they constituted the exposed group.

The control group comprised 46 administrative staff employees who were not working with computers. None of them used a computer for more than 1 h/day or lived near high voltage power lines or mobile base stations.

### Methods

All the participants completed a self administered questionnaire including questions about personal and present history, family history, past history, occupational history and if they lived near high voltage power lines or mobile base stations. Also the questionnaire included the following question to subjectively assess the sleep sufficiency; ”would you rate your total sleep time as sufficient or insufficient?

### Plasma melatonin and MDA assay

Venous blood samples of 5 mL each were collected from all the participants (at a fixed time (08:00 am) in EDTA tubes, then centrifuged and the serum was stored at –20°C until analysis.

The plasma melatonin assays were performed with a radioimmunoassay method described by Fraser *et al*.[16] using BioSource Kits (KIPL0800- BioSource International, Inc., Camarillo, California, U.S.). The assay procedure follows the basic principle of radioimmunoassay, involving competition between a radioactive and nonradioactive antigen for a fixed number of antibody binding sites. The amount of I ^125^ labeled antigen bound to the antibody is inversely proportional to the analyte concentration of the sample. When the system is in equilibrium, the antibody bound radioactivity is precipitated with a second antibody in the presence of polyethylene glycol. The precipitate is counted in a gamma counter. Quantification of unknown samples is achieved by comparing their activity with a reference curve prepared with known standards.[16] All assays were performed blinded to the subject’s case/control status and in a single series to avoid interassay variability.

MDA levels were measured by the thiobarbituric acid reactive substances method.[17]

### Statistical analysis

Data entry and analysis were accomplished using SPSS program version 16.0 (SPSS Inc., Chicago, U.S.) under Windows. The results were statistically analyzed using Chi-square test (χ^2^) and Fisher’s exact test for discrete data; and Student’s *t* test for parametric continuous variables. Mann–Whitney test was used for nonparametric continuous data. Pearson correlation coefficient was used to correlate data. Analysis of data was performed using the 0.05 significance level and the 0.01 high significance level.

## RESULTS

The demographic characteristics of both the groups of subjects are shown in Table 1.There was no statistically significant difference between the electronic equipment repairers (exposed group) and the controls with regard to matching variables, such as age, gender, residence, education level, smoking, and duration of employment (*P* > 0.05).

**Table 1:** Demographic characteristics of the study population

Variables	Exposed group (n = 50)	Controls (n = 46)	Tests of significance	*P* value
	Mean ± SD	Mean ± SD		
Age (y)	36.88 ± 9.10	34.43 ± 9.24	*t = 1.31	0.20
Duration of employment (y)	9.12 ± 6.69	9.22 ± 6.43	*t* = 0.07	0.94
Smoking				
Smoker	27 (54)	20 (43.50)	†χ^2^ = 1.06	0.32
Nonsmoker	23 (46)	26 (56.5)		
Education				
Secondary school	0 (0)	2 (4.30)	χ^2^ = 4.90	0.09
Technical graduation	27 (54)	31(67.40)		
University graduation	23 (46)	13 (28.30)		
Marital status				
Married	42 (84)	6 (13)	χ^2^ = 0.17	0.78
Single	8 (16)	40 (87)		
Residence				
Rural	6 (12)	12 (26.1)	χ^2^ = 3.12	0.06
Urban	44 (88)	34 (73.9)		

* Student’s *t* test;

† Chi-square test.

The mean levels of melatonin and MDA of the exposed group and controls are shown in Table 2. The mean level of serum melatonin in the electronic equipment repairers was lower than that of the controls with a high statistically significant difference (*P* < 0.01). Moreover, serum MDA mean level of the electronic equipment repairers was higher than that of the controls with a high statistically significant difference (*P* < 0.01).

**Table 2:** Comparison of melatonin and MDA mean levels between exposed group and controls

Parameters	Exposed Group (n = 50) mean ± SD	Controls (n = 46) mean ± SD	Tests of significance (Mann-Whitney test)	*P* value
Melatonin (pgm/mL)	16.35 ± 21.28	31.79 ± 29.34	2.79	0.00
	(0.00–76.01)	(0.50–78.81)		
MDA (mmol/mL)	3.58 ± 1.30	2.82 ± 0.93	2.52	0.01
	(1.79–5.91)	(1.48–4.80)		

MDA, malondialdehyde.

The prevalence of sleep insufficiency among exposed group and controls is shown in Table 3. Sleep insufficiency was more frequent among electronic equipment repairers (18.00%) in comparison to the controls (8.70%) but without a statistically significant difference.

**Table 3:** Prevalence of sleep insufficiency among exposed group and controls

Sleep insufficiency	Exposed group (n = 50) N (%)	Controls (n = 46) N (%)	Tests of significance (Chi-square test)	*P* value
Present	9 (18)	4 (8.70)	1.77	0.24
Absent	41 (82)	42 (91.30)		

The association between sleep insufficiency and melatonin, MDA, age, and duration of employment among the exposed group is shown in Table 4.Among the electronic equipment repairers, serum melatonin mean levels of the subjects complaining of sleep insufficiency (4.62 ± 3.63) were lower than those who were not complaining of sleep insufficiency (19.09 ± 23.24); but the statistical significance was border line (*P* < 0.06). However, MDA mean level, mean age, or mean duration of employment did not differ significantly (*P* > 0.05) according to the presence or absence of sleep insufficiency symptom among the electronic equipment repairers.

**Table 4:** Association between sleep insufficiency and melatonin, MDA, age and duration of employment among exposed group

Variable	Sleep insufficiency among Exposed group (N = 50)		Test of significance	*P* value
	Present (n = 9)	Absent (n = 41)		
MDA levels (mmol/ml)	2.94 ± 1.25	3.71 ± 1.29	†*Z* = 1.52	0.13
Melatonin levels (pgm/mL)	4.62 ± 3.63	19.09 ± 23.24	*Z* = 1.82	0.06
Age (y)	33.22 ± 7.76	37.68 ± 9.26	‡*t* = 1.34	0.19
Duration of employment (y)	7.06 ± 6.34	9.59 ± 6.75	*t* = 1.05	0.30

*Data are expressed as mean ± SD;

† Mann–Whitney test;

‡ Student’s *t* test.

MDA, malondialdehyde.

## DISCUSSION

Free radicals are very reactive and unstable molecular fragments that have an unpaired electron and they can produce new free radicals by means of chain reactions. These molecules, although formed as a result of normal biochemical processes, sometimes may be damaging and interact with all the macromolecules, including lipids, nucleic acids, and proteins. There are some mechanisms to neutralize their effects; 2 of them are nutritional and endogenous enzymatic antioxidant defenses that generally hold the production of free radicals and prevent oxidant stress and subsequently prevent tissue damage.[18,19]

On the other hand, melatonin is a powerful, endogenously produced scavenger of ROS, particularly the hydroxyl radical. Other ROS that melatonin scavenges include hydrogen peroxide, nitric oxide, peroxynitrite anion, hypochlorous acid, and singlet oxygen.[20,21] Moreover, melatonin increases the effectiveness of other antioxidants, such as superoxide dismutase, glutathione peroxidase, and catalase.[22,23] Melatonin has protective effects against ultraviolet and ionizing radiation and may also stimulate or activate DNA repair processes.[24]

However, the present study showed that electronic equipment repairers exposed to ELF-EMF had lower serum melatonin mean level in comparison to that of the controls with a high statistically significant difference (*P* < 0.01). Our findings are consistent with the hypothesis of a melatonin suppressing effect from magnetic field exposure.[25] Moreover, serum MDA mean level among electronic equipment repairers was higher than that of the controls (*P* < 0.01). These results denote that electronic equipment repairers were exposed to oxidative stress.

The metabolic processes that generate oxidants and antioxidants can be influenced by environmental factors, such as ELF-EMF. Increased ELF-EMF exposure can modify the activity of the organism by ROS, leading to oxidative stress.[26,27]

The free radical attack on unsaturated fatty acids of lipid structures may lead to lipid peroxidation, and may have damaging effects on proteins. Lipid peroxidation products, for example MDA, has been taken as a biomarker for oxidative stress in biological system.[20,28] Particularly susceptible to oxidative damage by free radicals are the polyunsaturated fatty acid acyl chains of phospholipids, which lead to lipid peroxidation. Uncontrolled lipid peroxidation is a toxic process resulting in the deterioration of biological membranes.[28–30] Ergüder *et al*.[31] found that MDA levels increased in the saliva samples obtained from volunteer subjects after computer use. They concluded that computer-released radiation causes changes in enzymatic antioxidant defense system, and leads to oxidant stress in saliva samples from subjects.

Concerning the prevalence of sleep insufficiency among the studied population, the present study showed that sleep insufficiency was more frequent among electronic equipment repairers exposed to ELF-EMF in comparison to the controls but without statistically significant difference.

Moreover, among the electronic equipment repairers, the mean level of serum melatonin in subjects complaining of sleep insufficiency (4.62 ± 3.63) was lower than those who were not complaining of sleep insufficiency (19.09 ± 23.24); but the statistical significance was border line (*P* < 0.06). However, MDA mean level, mean age, or mean duration of employment did not differ significantly according to the presence or absence of sleep insufficiency symptom.

Many authors reported that the consequences of sleep deprivation and sleepiness have been noted as a tremendous burden on our modern society and life, including an increase in mortality and morbidity, errors and accidents, absenteeism, a decrease in productivity, and the deterioration of personal and professional relationships.[32,33]

The present study supports the other studies that suggest that occupational exposure to ELF-EMF might have a detrimental effect on sleep quality.[34–37] Moreover, Yoshioka *et al*.[38] reported that workers who spent 6 h/day or longer on VDUs work tended to have sleep disturbances, especially problems in total sleep duration, and sleepiness during the day. It has been shown that sleep disturbances not only cause various illnesses but also seriously affect productivity in society.[39]

Early research concerning the relationship between melatonin and sleep was conducted on animals and its effects on gonadal maturation and circadian systems were examined. These early animal experiments provided evidence for chronobiological and sleep-inducing effects of melatonin, suggesting a role for this hormone in sleep and behavior in humans.[40]

The limitations of our study were (a) small sample size, and (b) we could not take night blood samples to assess melatonin at night nor could we take multiple samples to assess rhythmic changes of melatonin level according to the time of the day. Also, we need to reassess sleep quality more accurately in further studies.

## CONCLUSION

The electronic equipment repairers who were exposed to EMF had lower plasma melatonin mean level and higher MDA mean levels, indicating a state of oxidative stress. Moreover, exposure to EMF was associated with sleep insufficiency. Health education about the hazards of EMF, shortenings of EMF exposure time per day, and taking antioxidant vitamins should be done to ameliorate the oxidative effect of EMF on those workers.
